# Population pharmacokinetic modeling of dolutegravir/lamivudine to support a once-daily fixed-dose combination regimen in virologically suppressed adults living with HIV-1

**DOI:** 10.1128/aac.01504-23

**Published:** 2024-04-08

**Authors:** Hardik Chandasana, Rajendra Singh, Kimberly Adkison, Mounir Ait-Khaled, Teodora Pene Dumitrescu

**Affiliations:** 1 Clinical Pharmacology Modeling & Simulation, GSK, Collegeville, Pennsylvania, USA; 2 ViiV Healthcare, Durham, North Carolina, USA; 3 ViiV Healthcare Ltd., Brentford, United Kingdom; St. George’s, University of London, London, United Kingdom

**Keywords:** dolutegravir/lamivudine fixed-dose combination, population pharmacokinetic, HIV-1

## Abstract

A fixed-dose combination (FDC) of 50 mg dolutegravir and 300 mg lamivudine is indicated for the treatment of HIV-1 infection. This analysis aimed to characterize the population pharmacokinetics (PK) of dolutegravir and lamivudine based on data from a phase 3 study (TANGO) in virologically suppressed adults living with HIV-1 switching to dolutegravir/lamivudine FDC. These analyses included 362 participants who contributed 2,629 dolutegravir and 2,611 lamivudine samples collected over 48 weeks. A one-compartment model with first-order absorption and elimination parameterized by apparent oral clearance (CL/F), apparent volume of distribution (V/F), and absorption rate constant (Ka) described dolutegravir PK. Covariate search yielded body weight, bilirubin, and ethnicity as predictors of CL/F, and weight was predictive for V/F. The estimates of CL/F, V/F, and Ka were 0.858 L/h, 16.7 L, and 2.15 h^−1^, respectively. A two-compartment model with first-order absorption and elimination parameterized by CL/F, apparent intercompartmental clearance (Q/F), apparent central volume of distribution (V2/F), apparent peripheral volume of distribution (V3/F), and Ka described lamivudine PK. Covariate search yielded eGFR and race as predictors of CL/F, and weight was predictive for V2/F. The estimated parameter values were CL/F = 19.6 L/h, Q/F = 2.97 L/h, V2/F = V3/F = 105 L, and Ka = 2.30 h^−1^. The steady-state prediction suggested that the effect of covariates dolutegravir and lamivudine exposures was small (<20%) and not clinically relevant. Therefore, no dose adjustments are recommended based on these analyses. The results support the use of dolutegravir/lamivudine FDC in the treatment of HIV-1 infection in adults.

**CLINICAL TRIALS:**

This study is registered with ClinicalTrials.gov as NCT03446573.

## INTRODUCTION

Human immunodeficiency virus (HIV) is a retrovirus that primarily targets the CD4 receptor of T lymphocyte (CD4^+^ T) cells. Over time, HIV infection causes a reduction in host CD4^+^ T cells and immunological defects that can lead to acquired immune deficiency syndrome (AIDS) (1). The Joint United Nations Programme on HIV/AIDS estimated that 39.0 million people worldwide were living with HIV in 2022 and 29.8 million were receiving antiretroviral therapy (ART) (2). The introduction of three-drug regimen (3DR) ART in the mid-1990s has resulted in an increase in life expectancy of HIV-infected individuals approaching that of HIV-uninfected individuals. However, since treatment with ART is lifelong, there is growing concern over long-term toxicities, concomitant drug-drug interactions, and cost of 3DRs (3, 4). Two-drug regimens (2DRs) may help reduce long-term drug exposure, toxicity, and treatment costs while maintaining comparable efficacy to 3DRs (3, 4).

A fixed-dose combination (FDC) tablet of 50 mg dolutegravir and 300 mg lamivudine was recently approved by the United States (US) Food and Drug Administration and European Medicines Agency for the treatment of HIV type-1 (HIV-1) infection in adults in the US and adults and adolescents in the European Union (5, 6). Dolutegravir/lamivudine 2DR is indicated as a complete regimen for HIV-1 treatment in adults with no prior antiretroviral treatment history and no known substitutions associated with resistance to dolutegravir or lamivudine. Individuals should be tested for hepatitis B infection prior to or when starting therapy. Two phase 3, multicenter, double-blind, randomized, noninferiority studies (GEMINI-1 and GEMINI-2) evaluated the safety and efficacy of dolutegravir/lamivudine single-entity tablets as a complete 2DR for the treatment of HIV-1 infection in therapy-naïve adults compared to a 3DR of dolutegravir/tenofovir disoproxil fumarate/emtricitabine. These studies showed that dolutegravir/lamivudine 2DR was safe, well tolerated, and exhibited long-term noninferior efficacy in achieving virologic suppression compared to the 3DR (7, 8).

A separate clinical trial in healthy participants evaluated the bioequivalence in fasted state, food effect, and safety of a fixed-dosed formulation of 50 mg dolutegravir and 300 mg lamivudine compared to the same doses coadministered as single-entity tablets (9). This study showed that the FDC met bioequivalence standards for dolutegravir and lamivudine area under the concentration-time curve (AUC) and dolutegravir maximum concentration (Cmax). However, lamivudine Cmax was approximately 32% higher when administered with dolutegravir as an FDC compared to coadministration as single entities, suggesting a difference in the rate but not the extent of absorption for lamivudine in the fixed-dose formulation. The food effect for dolutegravir and lamivudine administered as an FDC tablet was found to be comparable to the known effects of food on the single entities. The results from this study supported the use of a fixed-dose formulation and demonstrated equivalency between the FDC tablet and the coadministered, single-entity tablets used in the GEMINI studies (9).

**Fig 1:**
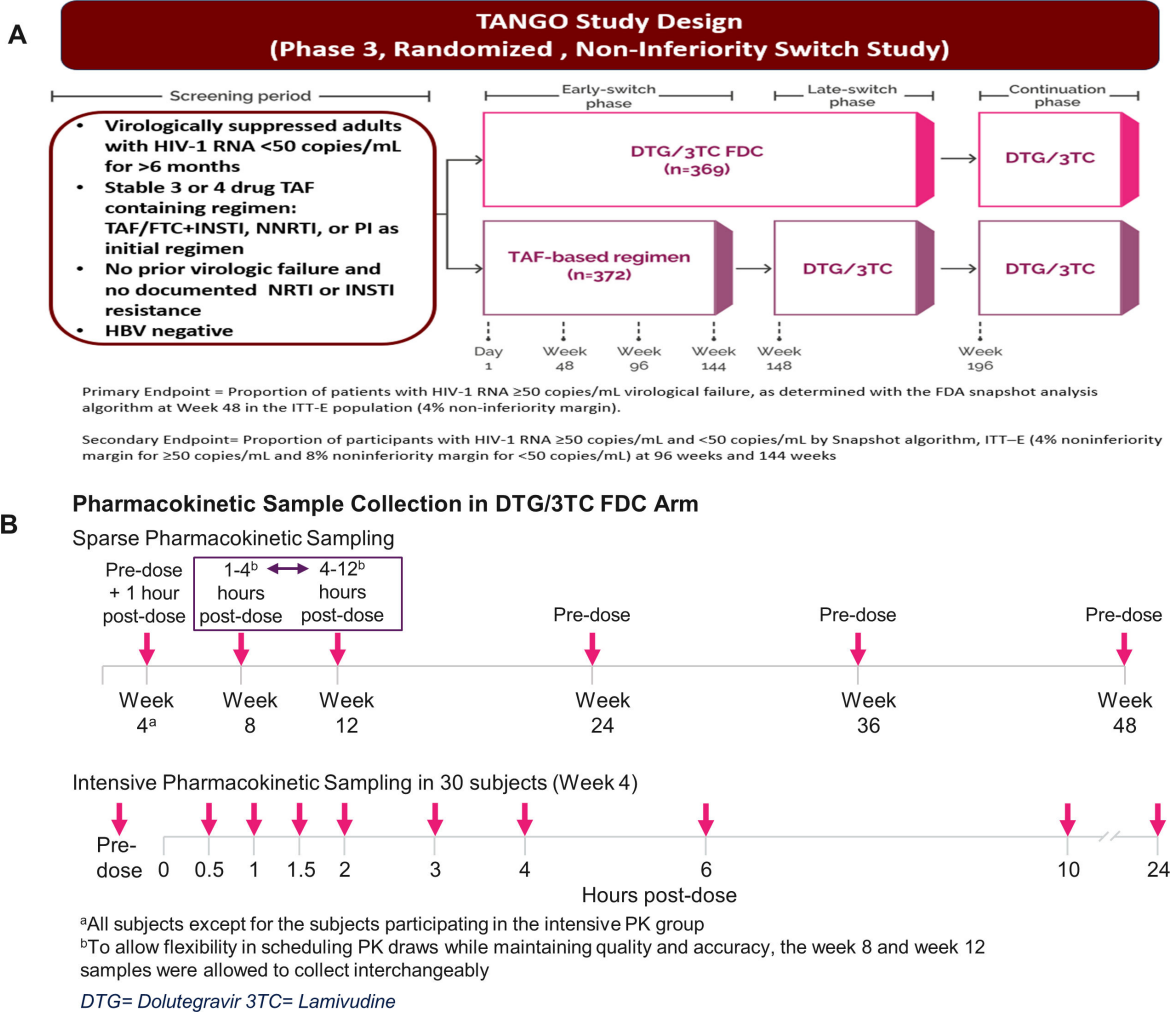
(**A**) TANGO study design and (**B**) PK substudy schematic.

TANGO was a phase 3, randomized, multicenter, parallel-group, noninferiority study evaluating the efficacy, safety, and tolerability of switching to dolutegravir/lamivudine FDC tablet in HIV-1-infected adults who are virologically suppressed (Fig. 1A). This study compared switching to once-daily dolutegravir/lamivudine FDC from a once-daily tenofovir alafenamide fumarate (TAF)-based regimen. A 48-week primary endpoint analysis of the phase 3 TANGO study showed that dolutegravir/lamivudine was noninferior in maintaining virologic suppression compared to a TAF-based regimen with no reported virologic failure or emergent resistance (10). A pharmacokinetic (PK) substudy in the dolutegravir/lamivudine FDC arm of the TANGO study was conducted to evaluate dolutegravir and lamivudine concentrations using sparse PK sampling collected in all participants and intensive PK sampling collected in a subgroup of participants to provide sufficient data for a thorough characterization of absorption phase (Fig. 1B). The objectives of this PK substudy, as reported herein, were to characterize the dolutegravir and lamivudine steady-state PK following once-daily oral administration of the dolutegravir/lamivudine FDC using population modeling methods to identify important determinants of variability and to provide post hoc steady-state exposure metrics (AUC0-τ, Cmax, and Cτ; τ = 24 hours post-dose) for both analytes. This analysis supported the regulatory approval of dolutegravir/lamivudine FDC in virologically suppressed adults with HIV-1.

## MATERIALS AND METHODS

### Study design and pharmacokinetic analysis

TANGO was a phase 3, randomized, open-label, noninferiority study designed to determine whether HIV-infected adults with virological suppression on a ≥three-drug TAF remain suppressed after switching to a 2DR of dolutegravir 50 mg/lamivudine 300 mg given once daily (10). Participants were randomized 1:1 to the dolutegravir/lamivudine regimen or current TAF, and antiviral activity was monitored over 48 weeks. In an intensive PK substudy, serial PK samples were collected in fasted state in a subset of 30 participants receiving dolutegravir/lamivudine at week 4 (pre-dose, 0.5, 1, 1.5, 2, 3, 4, 6, 10, and 24 hours post-dose). Sparse samples were obtained without regard to food from most dolutegravir/lamivudine-assigned participants at weeks 4 (pre-dose and 1 hour post-dose), 8 (1–4 hours or 4–12 hours post-dose), 12 (4–12 hours or 1–4 hours post-dose), and weeks 24, 36, and 48 (pre-dose). Plasma samples were analyzed for dolutegravir and lamivudine concentrations using validated analytical methods based on protein precipitation followed by UHPLC-MS/MS analysis with a TurboIonSpray interface and multiple reaction monitoring. The lower limit of quantification in plasma was 20 ng/mL for dolutegravir and 2.5 ng/mL for lamivudine (9).

### Modeling methods

Dolutegravir and lamivudine concentrations were modeled in NONMEM version 7.3 (ICON Development Solutions, Ellicott City, MD) using first-order conditional estimation method with interaction, and run management was conducted with Pirana version 2.9.0 (Certara, L.P., Princeton, USA). Post-processing, diagnostic plots, and covariate analysis were conducted in R version 3.2.5. There were only 0.19% dolutegravir and 0.11% lamivudine samples below limit of quantification (BLQ) in this study. Given the smaller number of samples, BLQ modeling methods were not attempted and excluded from analysis. Dolutegravir model fitting began using a previously described internally developed one-compartment linear model with first-order absorption, parameterized for the absorption rate constant (Ka), apparent clearance (CL/F), apparent volume of distribution (V/F), and absorption lag time (ALAG) (11). Weight, smoking status, age, and total bilirubin were predictors of CL/F, weight was a predictor of V/F, and gender was a predictor of bioavailability. Addition of an ALAG and inter-individual variability (IIV) on Ka were explored in the current model development but were not supported by the data and thus were not retained. Informed by a previous internally developed population PK model for twice-daily dosing of lamivudine (12), base lamivudine model development began with a one-compartment linear model with first-order absorption and weight, and total creatinine clearance (CrCL) was the predictor of CL/F. Various residual error models were also explored in this model development, including proportional, mixed proportional, and additive error, with or without an IIV term on the proportional residual error. Model selection was guided by goodness-of-fit plots, successful convergence, plausibility and precision of parameter estimates, and objective function value (OFV). Following identification of the base structural models, covariate hypotheses for each analyte were tested. The impact of covariates chosen that could affect dolutegravir [weight, age, sex, smoking status, albumin level, total bilirubin, serum creatinine, estimated glomerular filtration rate (eGFR), Centers for Disease Control and Prevention (CDC) HIV classification (1 vs 2 and 3), ethnicity, race, and concomitant medications] or lamivudine [age, race, sex, serum creatinine, eGFR, CDC HIV classification (1 vs 2 and 3), ethnicity, creatinine clearance, and concomitant medications] PK parameter estimates for CL/F and/or V/F was assessed visually in each drug’s respective model with the forward/backward approach. Covariates with a significant impact on estimated CL/F or V/F OFVs were added to base models to become full covariate models. The effect of a covariate was tested by centering at the population median value and using a power model with estimated exponent. Covariates were retained in the final full models if a significant impact on PK parameter OFV was observed after a backward elimination process of the full covariate models. For forward and backward selections, a significance level of 0.01 [corresponding to 6.63 points change in the OFV for 1 degree of freedom (d.f.)] and 0.001 (corresponding to 10.83 points change in the OFV for 1 d.f.), respectively, for covariate model development was used. Predictive performance of final models was evaluated by prediction-corrected visual predictive checks (pcVPCs) and bootstrap analysis.

### Simulation methods

The final dolutegravir and lamivudine models were used to compute individual steady-state AUC0-τ, Cmax, and Cτ values for dolutegravir and lamivudine following steady-state dosing of treatment-experienced participants in the TANGO study. Steady-state concentrations were predicted at 0, 1, 2, 3, 4, 6, 8, 12, and 24 hours following a once-daily dose of dolutegravir 50 mg/lamivudine 300 mg. AUC0-τ (linear up/log down trapezoidal rule), Cmax, and Cτ were calculated from predicted concentrations by noncompartmental analysis in R software. These post hoc estimates of exposure were divided and summarized by categorical and continuous covariates identified to significantly influence PK or by population quartiles. In addition, in order to predict the impact of covariates on dolutegravir or lamivudine exposure, simulations were performed within NONMEM 1,000 times per subject for a total of 361,000 (361 × 1,000) simulated profiles. All participants from the population PK analysis (361 participants with their own set of covariates) were used for the simulation. The full population was first divided into categories or quartiles for categorical or continuous covariates, respectively. The geometric mean of each trial was computed for each category or quartile and normalized by the median geometric mean of the overall population serving as a reference parameter.

## RESULTS

A total of 2,629 dolutegravir and 2,611 lamivudine samples from 362 participants were included in the population PK analysis. Participants were primarily male, White, and below 65 years old. HIV-A was the most common HIV classification in the analysis population. The majority (95%) of participants had a CDC HIV infection classification of 1, 2, or 3 at baseline (Table 1). Concomitant medication use fluctuated by study visit, but only metal cation-containing products (11%–13%) and cytochrome P450 (CYP) 3A inhibitors (17%–19%) were consistently used in >10% of population and included in the covariate analysis. The CYP3A4 inhibitors (weak/moderate/strong) were polled together for this analysis.

**TABLE 1:** Summary of demographics for participants included in dolutegravir and lamivudine population PK analysis^*a*^

Covariate	All participants (*N* = 362)
	Median [range] or *N* (%)
Age (years) at baseline	40 [20–74]
Weight (kg) at baseline	78.8 [50.2–153.0]
Height (cm) at baseline	176.5 [150.0–195.6]
BMI (kg/m^2^) at baseline	25.3 [17.4–47.1]
BSA (m^2^) at baseline	1.98 [1.51–2.77]
Total bilirubin (μmol/L) at baseline	8.00 [2.00–34.00]
Albumin (g/L) at baseline	45.0 [37.0–53.0]
AST (IU/L) at baseline	20.0 [11.0–141.0]
ALT (IU/L) at baseline	19.0 [7.00–145.0]
Serum creatinine (mg/dL) at baseline	0.96 [0.52–1.69]
Glomerular filtration rate (mL/min/1.73 m^2^)	99.0 [44.0–147.0]
CrCL (mL/min) at baseline	113.9 [56.3–355.9]
Sex: male	337 (93.1)
Race^*b*^	
White	291 (80.4)
Black or African American	49 (13.5)
Asian	13 (3.59)
American Indian or Alaskan	7 (1.93)
Native Hawaiian or other Pacific races	1 (0.28)
Other	1 (0.28)
Ethnicity	
Non-Hispanic or Latino	292 (80.7)
Hispanic or Latino	70 (19.3)
Smoker status	
Former	68 (18.8)
Never	180 (49.7)
Current	114 (31.5)
Hepatitis co-infection at baseline	
None	346 (95.6)
Hepatitis C only	16 (4.4)
Renal impairment	
Normal (>90 mL/min)	298 (82.3)
Mild (60–89 mL/min)	63 (17.4)
Moderate (50–59 mL/min)	1 (0.28)
CDC classification of HIV infection at baseline	
1	249 (68.8)
2	94 (26.0)
3	19 (5.25)

^*a*^ ALT, alanine aminotransferase; AST, aspartate aminotransferase; BMI, body mass index; BSA, body surface area; CDC classification based on description given in https://www.cdc.gov/hiv/statistics/surveillance/terms.html; GFR calculated using CKD-EPI method; CrCL calculated using Cockcroft-Gault equation.

^*b*^ One subject had an incorrect race reported in the eCRF and was excluded from population PK analysis.

### Dolutegravir PK model

The final model selected for dolutegravir was a one-compartment model with first-order absorption and elimination. Residual error was fit to a proportional residual error model, and IIV was included on CL/F and the magnitude of proportional error. Bilirubin and ethnicity decreased OFV by 14.265 and 16.635 points, respectively, and were identified as predictors of dolutegravir CL/F; weight reduced OFV by 79.62 points and was identified as a predictor of both CL/F and V/F (Table 2). These covariates were incorporated into the final dolutegravir PK model to estimate post hoc exposures. In Hispanic or Latino participants, CL/F was 15.6% lower compared to those of non-Hispanic or Latino ethnicity. Over the range of observed bilirubin levels (2–34 µmol/L) in the analysis population, CL/F ranged from 24% higher to 20% lower than that of a typical subject with a bilirubin level of 8 µmol/L. Over the range of weights included in the analysis (50–153 kg), CL/F ranged from 18% lower to 33% higher, and V/F ranged from 34% lower to 83% higher than for a 79-kg subject.

**TABLE 2:** NONMEM and bootstrap parameter estimates for the final dolutegravir and lamivudine population PK models^*a*^

Parameter (units)	NONMEM			Bootstrap	
	Mean estimate	%RSE	95% CI	Median estimate	95% CI
Final dolutegravir model					
CL/F (L/h)	0.858	1.9%	0.826, 0.890	0.858	0.826, 0.892
V/F (L)	16.7	2.73%	15.8, 17.6	16.7	15.8, 17.6
Ka (h^−1^)	2.15	10.4	1.72, 2.59	2.14	1.783, 2.753
CL/F~WT	0.427	16.1	0.293, 0.562	0.427	0.263, 0.593
V/F~WT	0.917	11.4	0.711, 1.12	0.914	0.713, 1.147
CL/F~bilirubin	−0.153	−23.4	−0.223, –0.0827	−0.154	−0.227, –0.8
CL/F~ethnicity	0.844	4.06	0.777, 0.911	0.842	0.776, 0.913
Inter-individual variability					
ω^2^_CL/F_	0.0682	8.85	26.6	0.067	0.056, 0.079
ω^2^_proportional error_	0.0567	22.0	24.2	0.056	0.033, 0.084
Residual variability					
σ^2^_prop_	0.341	2.73	N/A	0.341	0.322, 0.360
Final lamivudine model					
CL/F (L/h)	19.6	2.19	18.8, 20.5	19.6	18.7, 20.5
V2/F = V3/F (L)	105	3.92	96.9, 113	105	97.9, 112.7
Q/F (L/h)	2.97	6.44	2.59, 3.34	2.96	2.55, 3.35
Ka (h^−1^)	2.30	15.3	1.6, 2.99	2.27	1.84, 3.85
CL/F~eGFR	0.533	18.9	0.336, 0.731	0.536	0.330, 0.732
CL/F~race	0.789	6.32	0.691, 0.887	0.788	0.695, 0.889
Inter-individual variability					
ω^2^_CL/F_	0.0883	19.1	30.4	0.0864	0.0565, 0.124
BSC_CL/F~V2/F_	−0.0531	−33.1	N/A	−0.0501	−0.091, –0.0180
ω^2^_V2/F_	0.158	19.9	41.4	0.159	0.099, 0.238
ω^2^_proportional error_	0.247	17.2	52.9	0.247	0.169, 0.344
Residual variability					
σ^2^_prop_	0.359	3.84	N/A	0.358	0.333, 0.390

^*a*^ Pharmacokinetic parameter estimation for dolutegravir: CLi = θCL × (WT/79)^(CL/F~WT)^ × (BILI/8)^(CL/F~BILI)^ × (CL/F~ETHN)^ETHN^ × exp(η1), where ETHN is 0 for non-Hispanic/Latino, 1 for Hispanic/Latino; Vi = θV × (WT/79)^(V/F~WT)^; Kai = θKa; Fi = 1; inter-individual variability: %CV = SQRT[exp(ω^2^) – 1] × 100; Residual error: Y = IPRED × [1 + θ_prop error_ × exp(η4) × ε1], where: ε1(σ^2^) fixed to 1; pharmacokinetic parameter estimation for lamivudine: CLi = θCL × (WT/70)^0.75^ × (eGFR/99)^(CL/F~eGFR)^ × (CL/F~RACE)^RACE^ × exp(η1), where RACE is 0 for non-Black/African American (combined White, Asian, Alaskan Native, Hawaiian Native, and other), 1 for Black/African American; V2i = θV2 × (WT/70) × exp(η2); V3i = V2i; Qi = θQ × (WT/70)^0.75^; Kai = θKa; Fi = 1; inter-individual variability: %CV = SQRT[exp(ω^2^) – 1] × 100; residual error: Y = IPRED × [1 + θ_prop error_ × exp(η5) × ε1], where: ε1(σ^2^) fixed to 1; BILI, bilirubin; BSC, between-subject covariance; CI, confidence interval; CL/F, apparent oral clearance; ETHN, ethnicity; F, bioavailability; Ka, absorption rate constant; N/A, not applicable; ε1, residual error; i, individual subject parameter; ω^2^, variance of random effect; IPRED, individual predicted concentrations; Q/F, apparent distributional clearance; %RSE, percent relative standard error of the estimate; V/F or V2/F, apparent central volume of distribution; V3/F, apparent peripheral volume of distribution; WT, baseline weight (kg). Note: For the dolutegravir model, the reference population for CL/F is a 79-kg non-Hispanic/Latino subject with bilirubin of 8 μmol/L. The reference population for V/F is a 79-kg subject. For the lamivudine model, the reference population for CL/F is a 70-kg non-Black/African American subject with eGFR of 99 mL/min/1.73 m^2^. The reference population for V2/F is a 70-kg subject.

**Fig 2:**
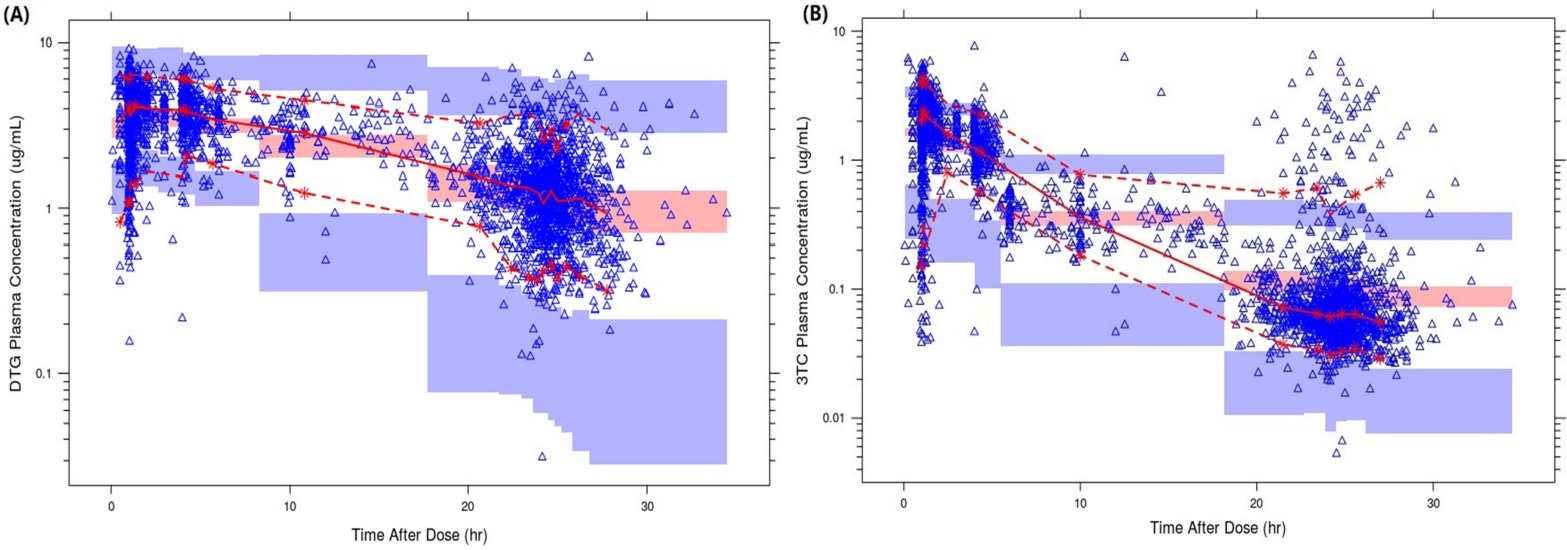
Prediction-corrected VPC plots of the final population pharmacokinetics models [(**A**) dolutegravir and (**B**) lamivudine]. Prediction-corrected visual predictive check plot. The solid red line represents the median observed plasma concentration, and the semitransparent red field represents a simulation-based 95% confidence interval for the median. The observed 5% and 95% percentiles are presented with dashed red lines, and the 95% confidence intervals for the corresponding model predicted percentiles are shown as semitransparent blue fields.

IIV on CL/F and the proportional residual error term were moderate at 26.6% and 24.2%, respectively, and all PK parameters were estimated with good precision as evidenced by relative standard error values <25%. Model diagnostics, as evaluated by goodness-of-fit plots (Fig. S1), bootstrap (Table 2), and normal prediction distribution error (NPDE) plots (Fig. S2), suggested adequate model fit and that predictive performance was acceptable for simulations. The median tendency of absorption phase was not captured effectively; however, overall, pcVPC plot indicates a good agreement between model predictions and dolutegravir individual observations (Fig. 2A).

Based on the final model, individual post hoc estimates of exposure (AUC0-τ, Cmax, and Cτ) were summarized based on the categorical covariates or by quartiles of exposures. The AUC0-τ, Cmax, and Cτ for individual participants increased with increasing bilirubin concentration, with a difference between quartiles with the lowest and highest bilirubin level groups of 12%, 13%, and 45%, respectively. Similarly, AUC0-τ, Cmax, and Cτ were 17%, 13%, and 35% higher in Hispanic/Latino participants, respectively, than in non-Hispanic/Latino participants. Participants of higher weight demonstrated lower predicted AUC0-τ and Cmax, with a difference between quartiles with the highest and lowest weight groups of 20% and 22%, respectively. Cτ values were similar across the weight groups.

### Lamivudine PK model

The final once-daily lamivudine model identified was a two-compartment model with first-order absorption and elimination with a proportional residual error model. There was a 126-point decrease in the OFV for the two-compartment model, and the diagnostic plots displayed a less pronounced bias compared to the one-compartment model , indicated that the 2-CMT model better described the lamivudine data. However, fitting the two-compartment model to the full data set resulted in difficulties estimating reliable values for V3/F. Therefore, model simplification was explored by fixing the V3/F value to either the estimated value from the model fit to the intensive PK subset (59.8 L) or to the value of V2/F in the subsequent runs, respectively. The two methods resulted in similar goodness-of-fit plots. However, V2/F = V3/F resulted in a lower OFV compared to intensive PK subset value by 21.8 points. Therefore, the V3/F = V2/F parameterization was carried forward. The IIV was included on CL/F and V2/F. Body weight was allometrically scaled on CL/F, apparent distributional clearance (Q/F), and V2/F with fixed exponents of 0.75 on CL/F and Q/F and 1.0 on V2/F.

The covariates eGFR and race (Black or African American vs all other races combined) were included in the final lamivudine model to estimate post hoc exposures as predictors of CL/F based on covariate model results (decreased OFV by 20.85 and 13.79 points, respectively). Specifically, over the range of observed eGFR values (44–147 mL/min/1.73 m^2^), CL/F of participants in the analysis population ranged from 35% lower to 24% higher than that of a typical subject with eGFR of 99 mL/min/1.73 m^2^, and CL/F of Black or African American participants was 21% lower than those of other races in the study. As compared to PK parameters for a typical 70-kg subject, over the range of observed weights (50–153 kg), CL/F and Q/F ranged from 22% lower to 80% higher in the analysis population, and V2/F or V3/F ranged from 29% lower to 119% higher.

The magnitude of IIV was low moderate on CL/F and moderate on both V/F and the proportional residual error term. Bootstrap values and 95% confidence intervals (CIs) were nearly identical to NONMEM estimates, and all parameters were estimated precisely (Table 2). Diagnostic plots (Fig. S3) demonstrated an adequate fit of the model to lamivudine data, though some bias was present at low concentrations, likely due to many samples having high pre-dose concentrations (~2%) , inconsistent with a pre-dose time of collection (Fig. S3). The reflection of the bias was more in lamivudine compared to dolutegravir model. This slight bias was similarly reflected through overprediction of trough concentrations in the pcVPC (Fig. 2B). The NONMEM and bootstrap estimates and 95% CI were almost identical for all parameters (Table 2), and NPDE plots displayed a close to normal distribution for the prediction error and did not reveal any particular bias in model predictions following once-daily FDC oral administration (Fig. S4). Overall, based on the goodness of fit, as well on the results from the bootstrap, pcVPC, and NPDE, the final model was deemed to have acceptable predictive performance for simulation purposes.

Individual predictions resulted in lamivudine AUC0-τ, Cmax, and Cτ values 15%, 10%, and 22% higher, respectively, in patients with eGFR <90 mL/min/1.73 m^2^ compared with those with eGFR >90 mL/min/1.73 m^2^. In Black/African American participants, AUC0-τ, Cmax, and Cτ were 24%, 11%, and 49% higher, respectively, than for White participants, with no major differences noticed in other race categories vs White participants. AUC0-τ and Cmax decreased with increasing weight, with a difference between the highest and lowest weight groups of 20% and 33%, respectively.

Final model-derived geometric means for the post hoc exposure steady-state parameters AUC0-τ (µg·h/mL) and Cmax (µg/mL) after once-daily dolutegravir/lamivudine FDC were 59.2 and 5.08 for dolutegravir and 14.1 and 2.50 for lamivudine, respectively (Table 3). These model-based post hoc PK parameters calculated in the overall population (361) were comparable to those observed in a subset of 30 participants with intensive PK data (Table S2). Thus, despite the slight overprediction of trough concentration in lamivudine pcVPC, the impact of this slight model misspecification was minimal on overall AUC0-τ, which is the best predictor of lamivudine antiviral activity. The level of residual unexplained variability (RUV) can be varied between participants due to sometime incorrectly collected dosing histories or compliance between participants. As mentioned above, ~2% of pre-dose observations were slightly higher compared to other pre-dose concentrations in this study and historical studies. This varying RUV magnitude suggests that the collected data from different participants have different information. Instead of completely removing them without any reason, we have added IIV on residual unexplained variability to better optimize both models (13).

**TABLE 3:** Comparison of 50 mg once-daily dolutegravir and 300 mg lamivudine exposure parameters in HIV-infected adults^*a*^

Analysis/study population (reference source)	AUC0-24 (µg·h/mL)	Cmax (µg/mL)	C24 (µg/mL)
Dolutegravir (50 mg once daily)			
TANGO population PK analysis; *n* = 361 (current analysis)	59.2 (57.4, 61.1)	5.08 (4.95, 5.22)	1.23 (1.15, 1.31)
Population PK analyses in HIV-infected treatment-naïve adults; *n* = 449 [SPRING-1 and SPRING-2 studies; (14)]	53.6 (52.3, 55.0)	3.67 (3.61, 3.74)	1.11 (1.06, 1.15)
Population PK analysis in HIV-infected, treatment-experienced adults; *n* = 371 (SAILING and VIKING studies)^*b*^	45.1 (43.4, 47.0)	3.26 (3.18, 3.36)	0.83 (0.77, 0.89)
Lamivudine (300 mg once daily)			
TANGO population PK analysis; *n* = 361 (current analysis)	14.1 (13.6, 14.6)	2.50 (2.43, 2.58)	0.089 (0.081, 0.098)
HIV-infected adults; *n* = 13 (15)^*c*^	16.6 (± 4.15)	3.46 (± 0.85)	0.146 (± 0.087)

^*a*^ AUC0-24 = area under the concentration-time curve (0–24 hours post-dose);C24 = concentration at 24 hours post-dose; QD = once daily; n = number of participants; PI = prescribing information; SD = standard deviation. Note: Unless otherwise stated, data are presented as geometric mean (95% CI).

^*b*^ Data shown for the overall population which includes participants on mild or moderate background ARV therapies as inducers (unpublished).

^*c*^ Parameters reported as mean (± SD) (CV%). C24 reported as Cmin.

### Simulations

**Fig 3:**
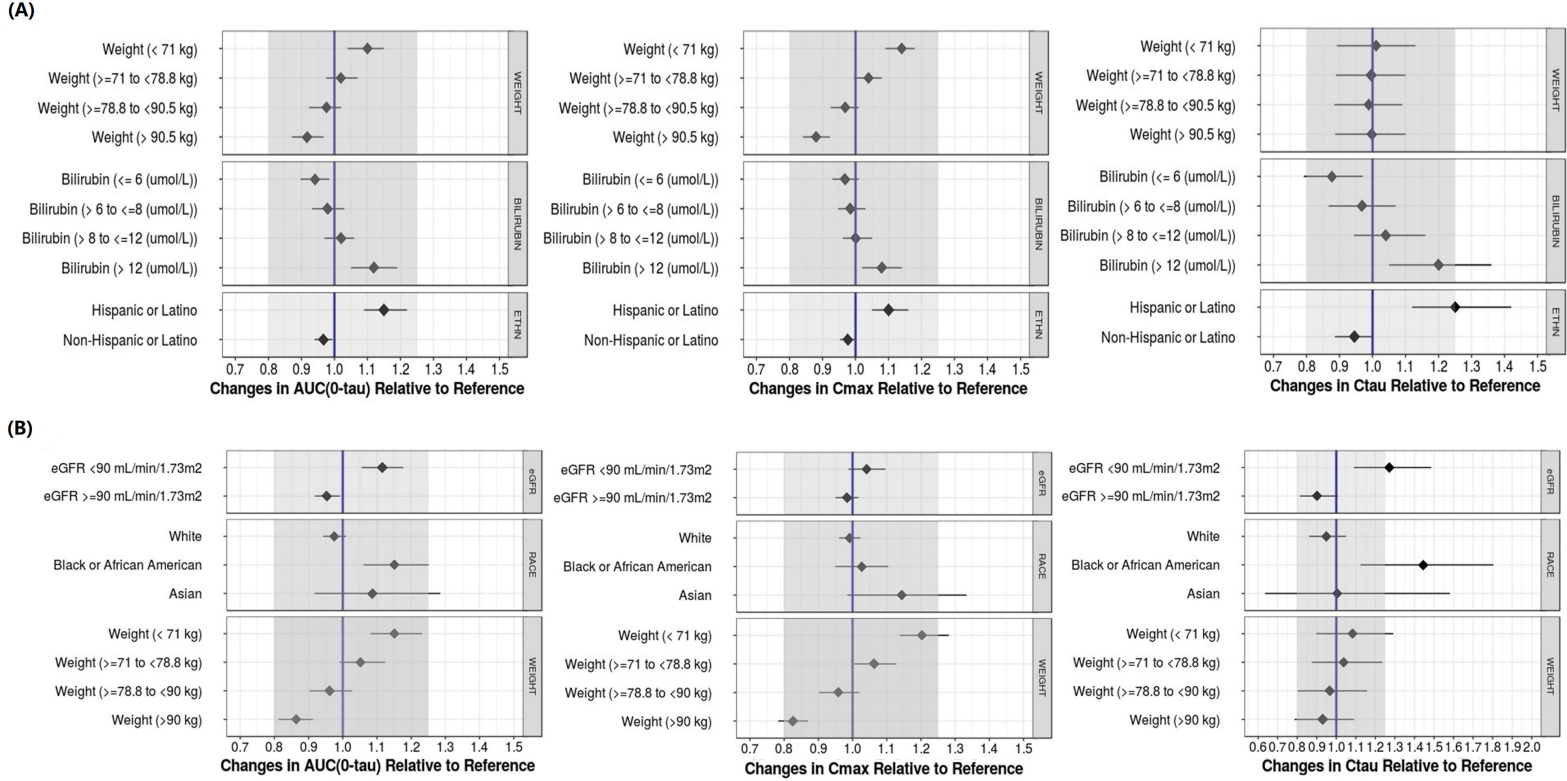
Predicted fold-change in steady-state once-daily dolutegravir (**A**) and lamivudine (**B**) AUC0-τ, Cmax, and Cτ relative to reference covariate category. Data from 1,000 simulated trials of *n* = 361 participants/trial separately for each drug. The line of unity (reference line) represents the median of 1,000 geometric means (from 1,000 simulated trials) for the overall population with. The median of geomeans of the overall population was used to normalize the geometric mean [AUC(0–τ), Cmax, Cτ] of each trial for each covariate category. Bars represent the medians, 2.5th, and 97.5th percentiles of the normalized geometric means for each covariate category. The shaded area represents bioequivalence criteria (0.8–1.25).

Forest plots depict the change in simulated dolutegravir exposure for subpopulations of varying weight, bilirubin levels, and ethnicity (Fig. 3A) and simulated lamivudine exposure for subpopulations of varying eGFR, race, and weight (Fig. 3B). Once-daily dosing of dolutegravir/lamivudine FDC demonstrated <20% magnitude of significant covariate effects compared to reference.

## DISCUSSION

In the present study, dolutegravir and lamivudine concentrations from a phase 3 study of once-daily dosing in virologically suppressed HIV-1 participants were modeled, and impactful covariates were identified. Similarly, to previous modeling analyses (11), dolutegravir concentrations were fit to a one-compartment linear model with first-order absorption and elimination. Effects of bilirubin and ethnicity on CL/F and weight on both CL/F and V/F were observed, though all effects were small and therefore considered to be not clinically meaningful. These results are consistent with previous dolutegravir analyses in HIV-infected treatment-naïve and treatment-experienced participants, and support that no dose adjustment of dolutegravir is necessary to account for bilirubin, ethnicity, or weight variability. Additionally, in this analysis, age, race, sex, smoking, albumin, Centers for Disease Control and Prevention HIV classification, serum creatinine, eGFR, alanine transaminase (ALT), aspartate transaminase (AST), prandial status (fasted state vs taken without regard to food), coadministration with CYP and UDP-glucuronosyltransferase inhibitors, or metal cation-containing products did not have significant impacts on dolutegravir PK.

In this analysis, lamivudine concentrations were best described by a two-compartment model with first-order absorption and elimination. This differs from previous lamivudine population PK analysis, which utilized a one-compartment model with first-order absorption and elimination (12). Of note, the previous lamivudine model was based on PK samples collected pre-dose and then at 0.5, 1, 2.5, 3.5, and 8 hours post-dose during twice-daily dosing. Therefore, the discrepancy in model structure likely originates from differences in the sampling strategy due to more samples being collected in the absorption, distribution, and elimination phase in the current study of once-daily dosing in relation to previous lamivudine studies.

The current analysis identified effects of renal function (eGFR) and race on CL/F. Renal function (creatinine clearance) was also identified as a predictor for CL/F in a previous analysis in HIV-infected participants (12). However, similarly to the previous analysis, all covariate effects were small and considered not clinically relevant; thus, no dose adjustment of lamivudine is recommended based on mild renal impairment or race. Lastly, weight was also identified as a predictor of CL/F and V/F in earlier analyses, though this effect was fixed to allometric exponents for this analysis. No effects of CDC classification, HCV co-infection, sex, age, ethnicity, smoking, ALT, AST, bilirubin, or prandial status on lamivudine PK were identified.

Modeled post hoc exposure steady-state parameters AUC0-τ (µg·h/mL) and Cmax (µg/mL) had geometric means of 59.2 and 5.08 for dolutegravir and 14.1 and 2.50 for lamivudine, respectively, after once-daily dolutegravir/lamivudine FDC administration. Overall, these parameters were generally consistent with values reported in previous pharmacokinetic studies in HIV-infected adults with the exception of Cmax of dolutegravir which was higher than that observed in historical data from treatment-naïve populations (Table 3) (11, 14–18). However, this difference is not considered clinically significant because the safety profile of dolutegravir/lamivudine FDC in the TANGO study is consistent with the safety profile of dolutegravir/lamivudine in treatment-naïve patients (7, 10).

Limitations of this study include a population consisting primarily of White (80%) and male (93%) participants. Additionally, the data set utilized for simulations was set to record samples at the subject level. This resulted in inclusion of any covariate correlations within that population; as such, differences between covariate categories may be confounded in this analysis due to imbalance of other covariates within these categories.

Simulations of steady-state dolutegravir and lamivudine exposure following fixed-dose dolutegravir/lamivudine administration demonstrated <20% magnitude of significant covariate effects compared to reference, signifying a lack of clinical significance and no need for dose adjustment based on these covariates (Fig. 3A and B). As such, the results from this modeling analysis support that once-daily dosing is an appropriate regimen for fixed-dose combination dolutegravir 50 mg/lamivudine 300 mg to treat HIV-1 infection.

## Data Availability

Anonymized individual participant data and study documents can be requested for further research from www.clinicalstudydatarequest.com.

## SUPPLEMENTAL MATERIAL

Supplemental material aac.01504-23-s0001.pdf Tables S1 and S2 and Figures S1 to S4.

ASM does not own the copyrights to Supplemental Material that may be linked to, or accessed through, an article. The authors have granted ASM a non-exclusive, world-wide license to publish the Supplemental Material files. Please contact the corresponding author directly for reuse.
