# Supplementary material for: Population pharmacokinetic modeling of dolutegravir/lamivudine to support a once-daily fixed-dose combination regimen in virologically suppressed adults living with HIV-1
Source: Antimicrob Agents Chemother. 2024 Apr 8;68(5):e01504-23. doi: 10.1128/aac.01504-23 (PMC11064544; doi:10.1128/aac.01504-23)

SUPPLEMENTARY INFORMATION

Table S1. Covariate exploration for the dolutegravir and lamivudine population PK analysis.

Covariate Type	Covariate	Categories	Evaluated in Dolutegravir Model	Evaluated in Lamivudine Model
Continuous	Age	NA	X	X
	Weight		X	X
	Total bilirubin		X	X
	CrCL		X	X
	eGFR		X	X
	Albumin		X	X
	SCR		X	X
	AST		X	X
	ALT		X	X
Categorical	Sex	Male<br>Female	X	X
	Race	White<br>Black<br>Asian<br>American Indian or Alaskan Native<br>Native Hawaiian or Other Pacific races<br>Other races	X	X
	Ethnicity	Hispanic or Latino<br>Non-Hispanic or Latino	X	X
	Smoking status	Never smoked	X	X

		Current smoker<br>Former smoker		
	Prandial status	Fed<br>Fasted<br>Information missing	X	X
	HCV coinfection	No<br>Yes	X	
	CDC classification of HIV	A<br>B<br>C	X	X
	Metal cation-containing vitamin/mineral supplements[a]	No<br>Yes	X	
	CYP3A inhibitors[a]	No<br>Yes	X	
	UGT inhibitors[a]	No<br>Yes	X	
	PGP inhibitors	No<br>Yes	X	X
	OCT inhibitors[b]	No<br>Yes	X	X

ALT, alanine aminotransferase; AST, aspartate aminotransferase; CDC, Centers for Disease Control and Prevention; CrCL, creatinine clearance; CYP, cytochrome P450; eGFR, estimated glomerular filtration rate; HCV, hepatitis C virus; HIV, human immunodeficiency virus; NA, not applicable; OCT, organic anion transporter; PGP, P-glycoprotein; PK, pharmacokinetics; SCR, serum creatinine; UGT, uridine diphosphate glucuronosyltransferase

[a]Excluded from testing covariates in the lamivudine model due to no known impact of drug on lamivudine PK.

Table S2 Summary of Plasma Dolutegravir and Lamivudine Steady-state PK Parameters Calculated for Participants with Serial PK Collected at Week 4

PK Parameter	Dolutegravir (n=30)	Lamivudine (n=30)
$AUC_{(0-\tau)}$ (μg*h/mL)	60.5 (45.3)	13.7 (42.2)
C_{max} (μg/mL)	4.56 (35.1)	2.58 (32.6)
C_{τ} (μg/mL)	1.27 (91.3)	0.098 (200.4)

The PK parameters were calculated suing non-compartmental (NCA) PK analysis method.
Data are presented as geometric mean (CVb%).

Figure S1. Goodness of fit plots for the final dolutegravir model. Circles are observed data, red line is loess smoother, black line is line of unity.

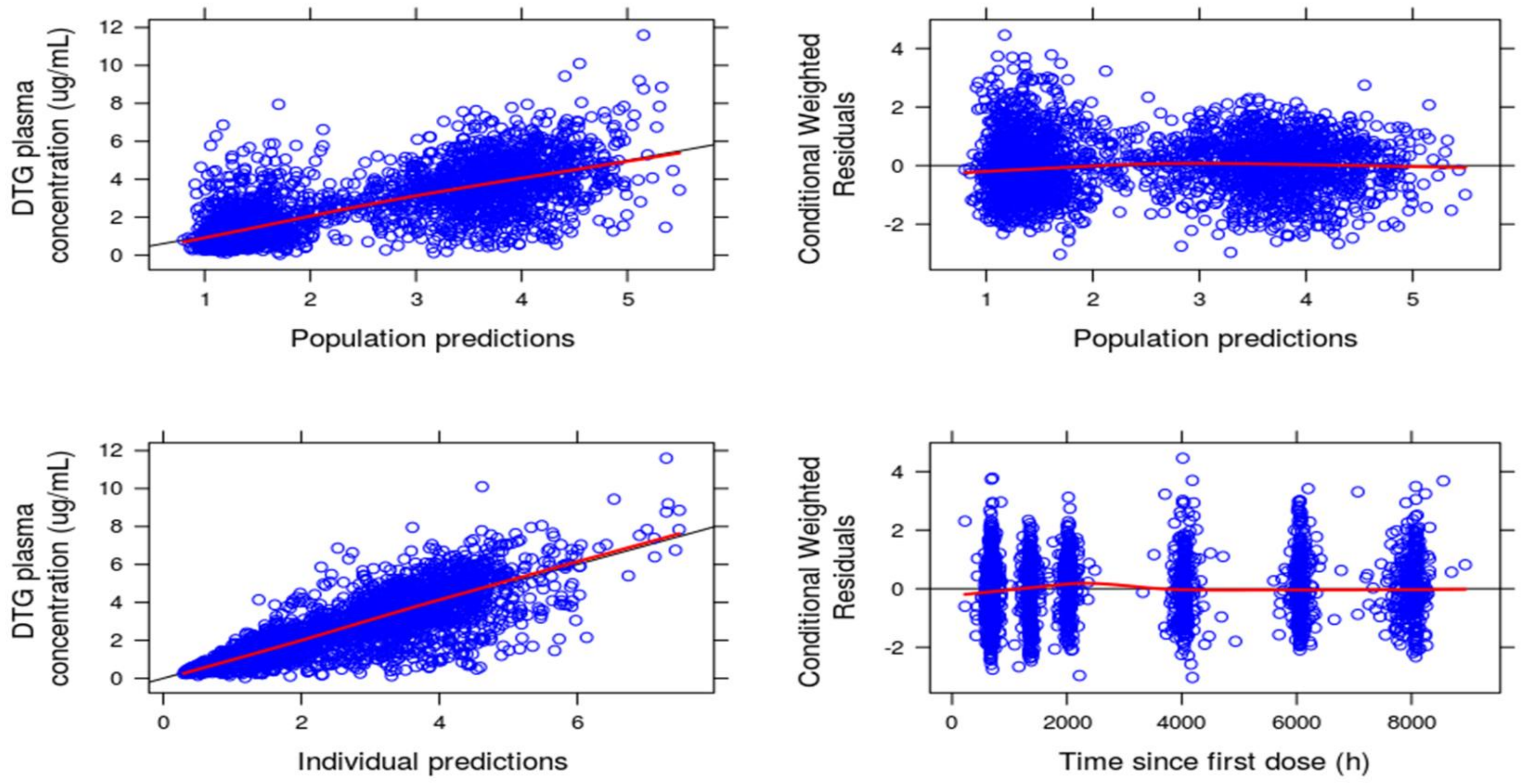

Figure S2. NPDE plots for final dolutegravir model. Sample, sample NPDE quartiles; NPDE, normal prediction distribution error; PRED, predicted individual concentrations; Theoretical, theoretical quartiles.

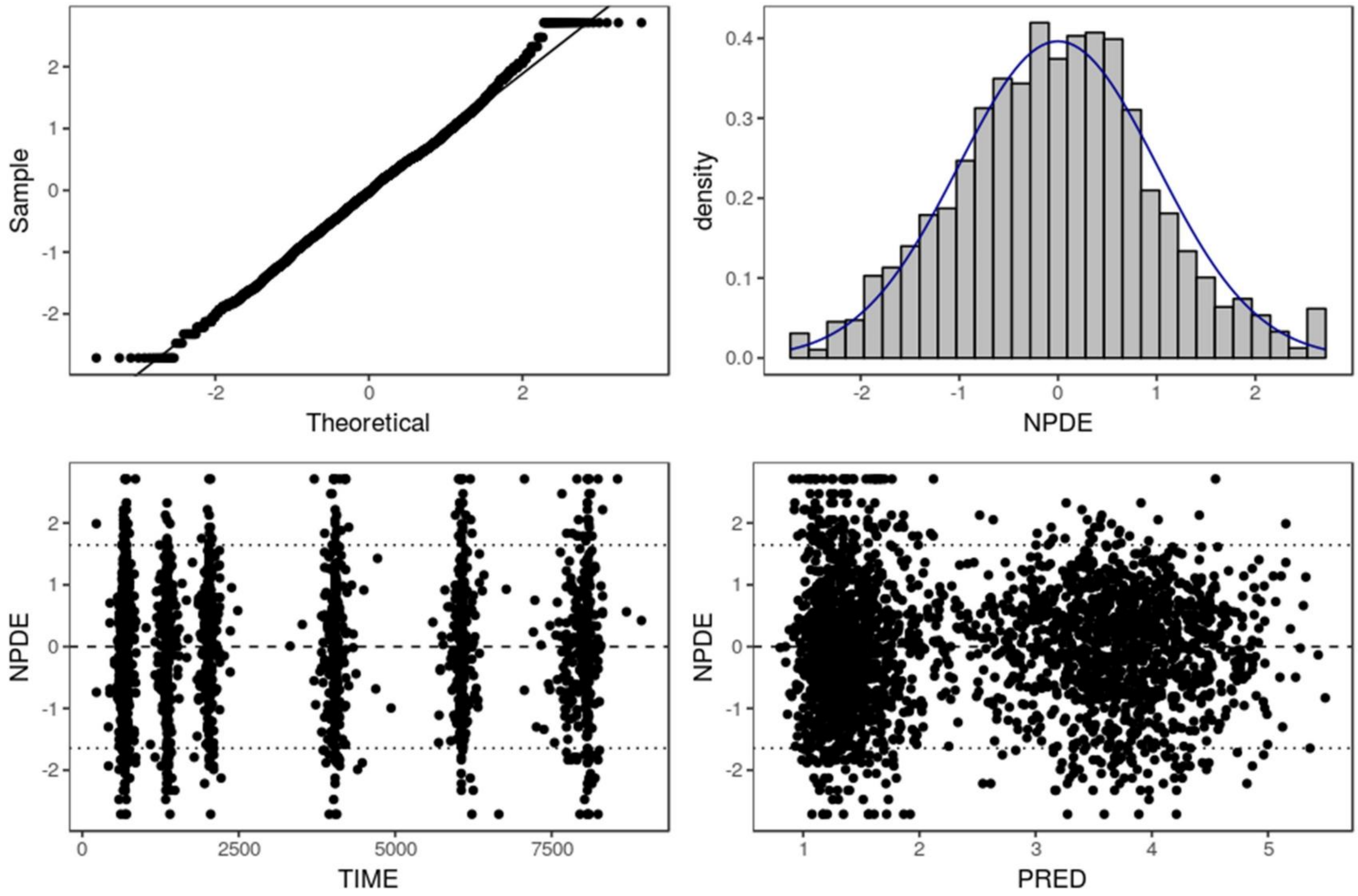

Figure S3. Goodness of fit plots for the final lamivudine model. Circles are observed data, red line is loess smoother, black line is line of unity.

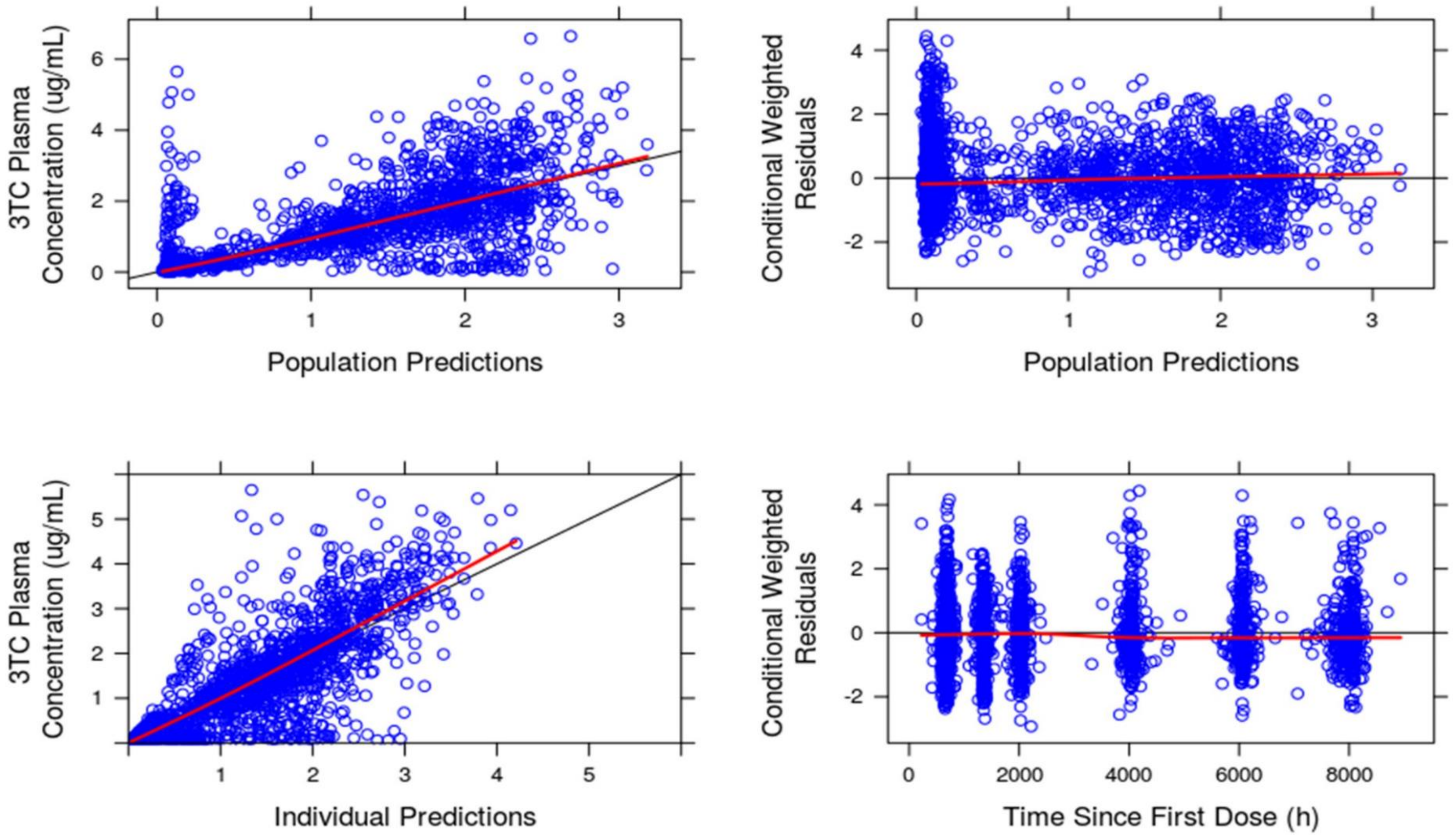
3TC Plasma Concentration (ug/mL)
Population Predictions
Conditional Weighted Residuals
Population Predictions
3TC Plasma Concentration (ug/mL)
Individual Predictions
Conditional Weighted Residuals
Time Since First Dose (h)

Figure S4. NPDE plots for final lamivudine model. Sample, NPDE; NPDE, normal prediction distribution error; PRED, predicted individual concentrations.

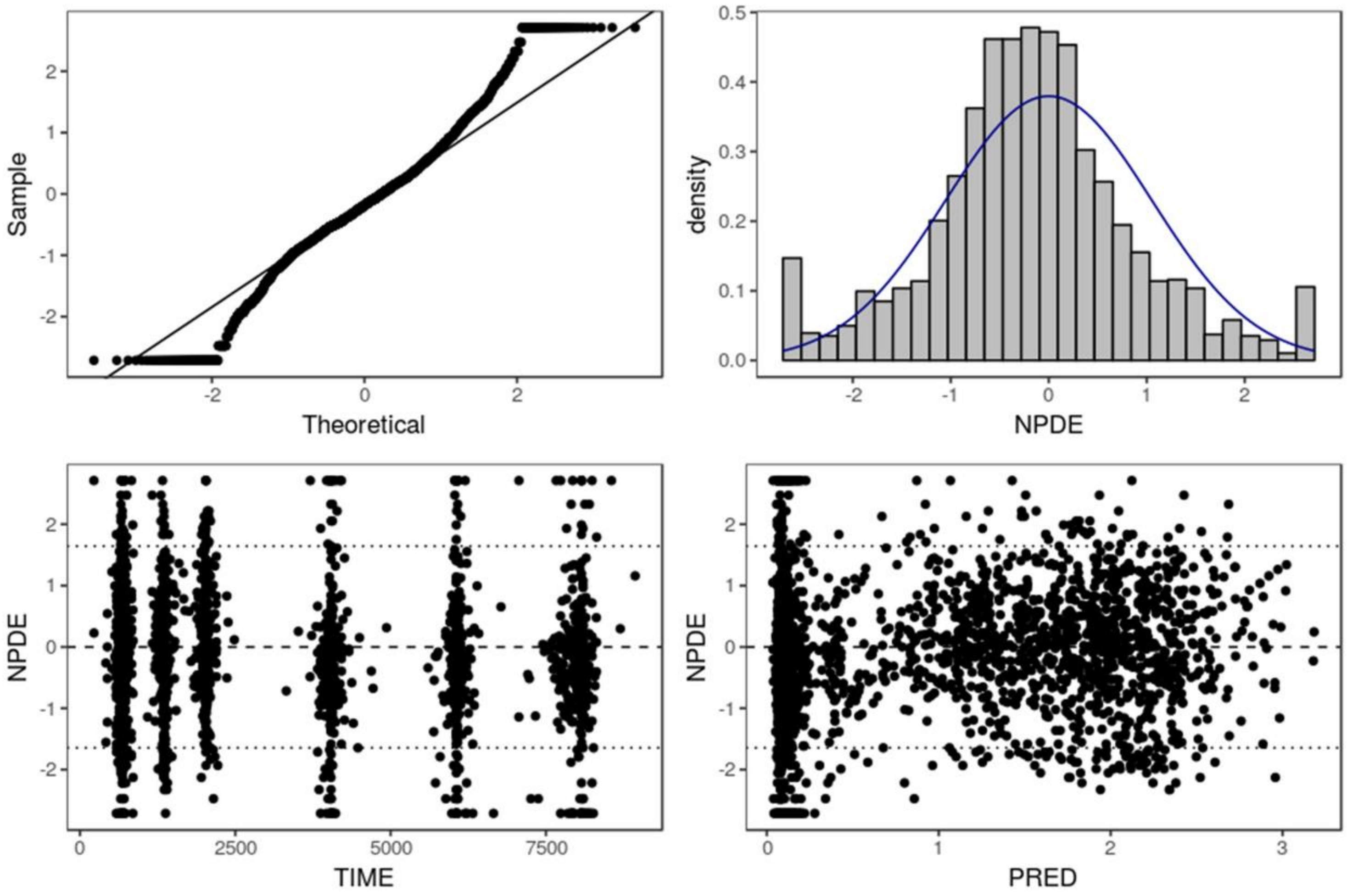

Supplement: Supplemental material — Tables S1 and S2 and Figures S1 to S4. [file aac.01504-23-s0001.pdf]
